# Finding the elusive balance between reducing fatigue and enhancing education: perspectives from American residents

**DOI:** 10.1186/1472-6920-14-S1-S11

**Published:** 2014-12-11

**Authors:** John Hanna, Daniel Gutteridge, Venu Kudithipudi

**Affiliations:** 1 Radiology Department, McLaren Flint/ Michigan State University, Flint, Michigan, USA; 2 Internal Medicine Department, Division of Pulmonary and Critical Care, Indiana University School of Medicine, Indianapolis, Indiana, USA; 3 Radiology Department, Duke University, Durham, North Carolina, USA

## Abstract

Duty hour restrictions for residency training were implemented in the United States to improve residents’ educational experience and quality of life, as well as to improve patient care and safety; however, these restrictions are by no means problem-free. In this paper, we discuss the positive and negative aspects of duty hour restrictions, briefly highlighting research on the impact of reduced duty hours and the experiences of American residents. We also consider whether certain specialties (e.g., Emergency Medicine, Radiology) may be more amenable than others (e.g., Surgery) to duty hour restrictions. We conclude that feedback from residents is a crucial element that must be considered in any future attempts to strike a balance between reducing fatigue and enhancing education.

## Introduction

The death of Libby Zion in March 1984 in a Manhattan hospital while under the care of fatigued residents was the tipping point that led to important reforms to the training and supervision of medical residents in the United States [1]. These reforms were undertaken with three things in mind: resident educational experience and quality of life, as well as patient safety. In 1989, New York State began to restrict working hours for medical residents. This example was followed by other states until, in 2003, the Accreditation Council for Graduate Medical Education (ACGME) began to enforce limited work hours for residents. Specifically, residents were limited to 80 hours per week, with no more than 30 consecutive hours of work and a 10-hour break after a 24-hour shift. As well, it was mandated that interns had to be supervised at all times. More recently, in 2011, the ACGME implemented a duty hour restriction for interns that saw the maximum call length reduced to 16 hours. This paper provides an analysis of the pros and cons of these duty hour reforms as debated in recent literature.

### Educational experience

There is no question that residency is the catalyst that transforms knowledge into competency and skills into experience. Attending physicians draw upon their residency experience – including the knowledge gained at the bedside and from the example of supervisors and peers – throughout their practice years.

It could be argued that the ultimate residency experience would be to live in the hospital. In fact, historically, residency implied exactly that, and until relatively recent times residents could be on-site for as long as a week at a time. However, these types of schedules were eliminated because of the social and psychological effects on residents and their families. Today, we sometimes send residents home from the hospital against their will. Have we gone too far?

Opinions on the appropriate level of duty hour restriction are mixed [2,3]. While residents agreed that the 80-hour limit was important, the more recently imposed 16-hour shift limit for interns has been met with some criticism [2].

Arguments against the 16-hour shift limit focus on the possible negative implications for continuity of care and for residents’ opportunities for learning. Specifically, concerns have been raised about reduced exposure to overnight events (codes and emergent procedures) [4,5]. However, the results of one study that analyzed resident knowledge and test scores at one 14-hour shift pilot site showed no decline in either area with the introduction of duty hour restrictions [6,7].

Although technology, including simulations, taped lectures, and other electronic resources, can help to supplement residents’ education, it cannot replace the real-life experience of repairing an aortic aneurysm or delivering a breech baby [4,8]. Working on real patients creates a level of intensity – both physical and emotional – that cannot be fabricated.

With that said, duty hour regulation has some clear benefits that should not be ignored. Certain fields, such as Emergency Medicine and Radiology, lend themselves to the new shift schedules. Residents in these fields tend to have more autonomy and opportunities to hone their skills because there are fewer staff members on-site [9]. This is particularly true with the introduction of off-hour shifts, which provide additional opportunities for exposure to complex cases (e.g., trauma, stroke). As well, the new duty hours tend to better mimic the post-training work schedules of attending physicians.

### Patient care and safety

The impact of duty hour changes on patient care and safety is difficult to assess [10]. On the one hand, Mann and Danz showed a significant improvement in the miss rate of radiological diagnoses (1.0 versus 1.7 misses per shift) when residents covered a 9-hour night versus a 23-hour shift [9]. In addition, Privette and colleagues reported a decrease in mortality rates (1.9 versus 1.0) after the implementation of night floats on surgical services [11].

On the other hand, Landrigan and colleagues showed an increase in the number of serious medical errors in intensive care units when shift lengths were decreased from 24 hours to 16 hours [12]. As well, Buskowski showed an increase in cesarean section deliveries with the introduction of night floats [5].

Although the studies described above focused on objective metrics, it is also important to consider residents’ opinions of the impact of restricted duty hours on patient care and safety. Three separate studies [13-15] that surveyed residents’ errors all showed significantly fewer self-reported errors across orders (i.e., nursing, laboratory tests, and medication) with the introduction of duty hour restrictions. However, a study that focused on a surgical service showed that 76% of residents agreed that implementing a night float had negatively affected the continuity of patient care [4]. Other studies that examined the opinions of residents, faculty, and support staff (including nurses) have provided a wide range of perspectives, even within the same setting [16].

While there will always be mixed opinions about duty hour restrictions, as a whole, the objective metrics and residents’ perspectives reported in the literature support the use of shorter shifts [10].

### Resident’s quality of life

One of the main goals of restructuring residency duty hours was to improve residents’ quality of life by enabling them to achieve a better balance between work and home [17]. Historically, higher rates of car accidents (a 16.2% increase after a 24-hour shift), needlestick injuries (a 50% increase after 29 consecutive work hours), and symptoms of burnout (a 55% increase in burnout at the end of the intern year) have been attributed to resident exhaustion [18-20]*.* The hope was that a reduction in resident duty hours would remedy these and other problems.

Despite this, Institute of Medicine (IOM) trials of 16-hour duty periods show that quality of life both at home and at work was reduced with the introduction of the duty hour restrictions [7]. Although the number of hours slept per week during the IOM trials increased marginally from 51 to 53.4, at the same time, “end of shift sleepiness” and “post-call sleepiness” rates increased [7]. Across the board, residents participating in the pilot programs reported higher levels of emotional exhaustion and lower levels of personal accomplishment, both of which are indicators of burnout [3,21]. Workload compression requires residents to scramble to see and understand the same number of cases in a shorter period of time, leaving them more stressed and unsure of their abilities [22]. The duty hour restrictions, although designed to improve residents’ quality of life, have been shown to have few positive effects, such that 58% of residents in an IOM trial were dissatisfied with the 16-hour duty rule [23].

Resident stress levels will remain elevated with the unbalanced new approach to duty hours, with levels of anxiety and depression remaining unchanged [24]. In fact, in one study, stress increased among upper-level Internal Medicine residents with the introduction of the 16-hour shift because of the subsequent increase in their workload [24].

## Conclusion

Medical education is constantly evolving and requires careful monitoring and responsive adaptation to changes in medicine and society. Although we are centuries removed from apprenticeship training, transfer of skills and knowledge today still relies on repetition. The difficulty of balancing training, patient safety, and resident quality of life continues to test the ingenuity of medical educators across the United States. Tipping the scales either way may result in more cases like Libby Zion’s, whether as a result of the fatigue of overworked interns or, potentially, and even more frighteningly, the under-preparation of physicians trained within a system of reduced duty hours. It is clear that a balance must be struck between reducing fatigue and enhancing education. While some of the new rules have been shown to be helpful (e.g., increased supervision), the radical decrease in hours has not. The correct balance can be found only by listening to resident feedback. They are the people in the trenches who are continually adapting to these duty hour changes. In our opinion, we are moving in the right direction; however, that elusive balance has yet to be found.

## Authors’ contributions

The authors contributed equally to the preparation of this article.

## Competing interests

The authors have no competing interests to declare.

## Declarations

Resources and secretariat support for this project was provided by the Royal College.

This article has been published as part of *BMC Medical Education* Volume 14 Supplement 1, 2014: Resident duty hours across borders: an international perspective. The full contents of the supplement are available online at http://www.biomedcentral.com/bmcmededuc/supplements/14/S1. This article was submitted in 2011 and peer reviewed in 2012. Final acceptance for publication as part of this supplement was in 2014.

Publication of this supplement was supported and funded by the Royal College of Physicians and Surgeons of Canada. The funding agency played no role in the design, in the collection, analysis and interpretation of data; in the writing of the manuscripts; and in the decision to submit the manuscript for publication. The articles have been through the journal's standard peer review process for supplements. The Supplement Editors declare that they have no competing interests.
